# Study of Emissions from Domestic Solid-Fuel Stove Combustion in Ireland

**DOI:** 10.1021/acs.energyfuels.0c04148

**Published:** 2021-02-26

**Authors:** Anna Trubetskaya, Chunshui Lin, Jurgita Ovadnevaite, Darius Ceburnis, Colin O’Dowd, J. J. Leahy, Rory F. D. Monaghan, Robert Johnson, Peter Layden, William Smith

**Affiliations:** † Department of Chemical Sciences, University of Limerick, Limerick V94 T9PX, Ireland; ‡ State Key Laboratory of Loess and Quaternary Geology, Key Laboratory of Aerosol Chemistry and Physics, Institute of Earth Environment, Chinese Academy of Sciences, Xi’an 710061, China; § CAS Center for Excellence in Quaternary Science and Global Change, Chinese Academy of Sciences, Xi’an 710061, China; ∥ School of Physics and Centre for Climate and Air Pollution Studies, Ryan Institute, National University of Ireland Galway, University Road, Galway H91 R8EC, Ireland; ⊥ MaREI, the SFI Research Centre for Energy, Climate and Marine, Galway P43 C573, Ireland; # School of Engineering and Ryan Institute, National University of Ireland Galway, Galway H91 TK33, Ireland; ∇ Arigna Fuels, Arigna Carrick-on-Shannon Co., Roscommon N41 E527, Ireland; ○ Department of Mechanical Engineering, University College Dublin, Dublin, Ireland

## Abstract

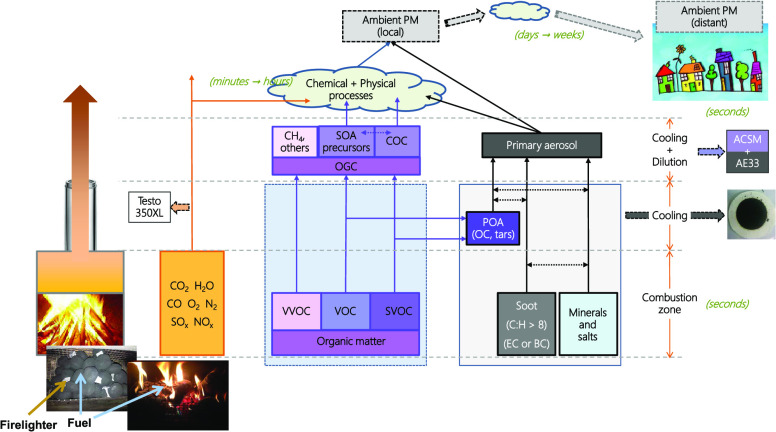

Solid-fuel stoves are at the heart of many homes not only in developing nations, but also in developed regions where there is significant deployment of such heating appliances. They are often operated inefficiently and in association with high emission fuels like wood. This leads to disproportionate air pollution contributions. Despite the proliferation of these appliances, an understanding of particulate matter (PM) emissions from these sources remains relatively low. Emissions from five solid fuels are quantified using a “conventional” and an Ecodesign stove. PM measurements are obtained using both “hot filter” sampling of the raw flue gas, and sampling of cooled, diluted flue gas using an Aerosol Chemical Speciation Monitor and AE33 aethalometer. PM emissions factors (EF) derived from diluted flue gas incorporate light condensable organic compounds; hence they are generally higher than those obtained with “hot filter” sampling, which do not. Overall, the PM EFs ranged from 0.2 to 108.2 g GJ^–1^ for solid fuels. The PM EF determined for a solid fuel depends strongly on the measurement method employed and on user behavior, and less strongly on secondary air supply and stove type. Kerosene-based firelighters were found to make a disproportionately high contribution to PM emissions. Organic aerosol dominated PM composition for all fuels, constituting 50–65% of PM from bituminous and low-smoke ovoids, and 85–95% from torrefied olive stone (TOS) briquettes, sod peat, and wood logs. Torrefied biomass and low-smoke ovoids were found to yield the lowest PM emissions. Substituting these fuels for smoky coal, peat, and wood could reduce PM_2.5_ emissions by approximately 63%.

## 1 Introduction

Emissions from domestic solid-fuel combustion in Ireland have been declining steadily since the introduction of the Air Pollution Act in 1987, with a rapid reduction in particulate matter and sulfur pollution in Dublin following the introduction of a ban on bituminous coal in 1990.^1,2^ Subsequent amendments to the legislation to include limits on particulate matter emissions and sulfur content, and the introduction of low-smoke zones, have been driven by the serious health risks associated with emissions from solid-fuel combustion. Nonetheless, emissions from the residential heating sector continue to impact significantly the local air quality, with the bulk of emissions arising from combustion of bituminous coal and peat.^3,4^

In Ireland, official data indicates that the mix of solid fuels for domestic heating is dominated by peat, followed by bituminous coal and manufactured ovoids, with biomass accounting for less than 10% of supply on an energy basis. However, the amount of nontraded wood and sod peat used in the residential sector is highly uncertain.^5^ A more detailed analysis of the nontraded sector suggests that, in a worst-case scenario, wood might account for 75 ktoe (13%) of final energy consumption in the residential sector.^6^ Combustion of bituminous coal is currently restricted to rural areas and small towns, with a nationwide ban anticipated.^7,8^ Replacing bituminous coal with manufactured briquettes derived from fossil fuels can reduce emission of PM from the residential heating sector but has little impact on CO_2_ emissions. Recent legislation therefore promotes “slow renewable”, “low-carbon”, or “carbon-neutral” biomass-based fuels for domestic heating.^9^ However, the potential for biomass combustion to emit high levels of PM_10_ and PM_2.5_,^3^^3^ of volatile organics, and of carbon monoxide^10,11^ remains a concern. In general, therefore, burning of solid fuel in traditional stoves and fireplaces can lead to emission of many pollutants, including PM_2.5_, black carbon (BC), brown carbon, toxic elements, CO, NOx, and SO_2_.^12−14^^12−14^ Of particular concern are emissions released from open fires and old stoves, especially when combined with unsuitable fuels like unseasoned wood or household waste.^15,16^

Drying of solid biomass fuels is known to reduce pollutant emissions during combustion. Moisture content of wood logs can be reduced from ≈45% to below 25% by long-term storage, or “seasoning”.^17,18^ Forced heat drying at temperatures below 150 °C can reduce moisture levels to less than 15%, albeit with attendant financial and energy costs.^19^ The next level of thermal treatment is torrefaction, a mild pyrolysis process. Torrefaction contributes concurrently to dehydration, deoxygenation, partial degassing, and structural changes through breaking hemicellulose, lignin, and cellulose chains at elevated temperatures. These changes yield a fuel with increased calorific value and improved physiochemical properties. Studies suggest that combustion of torrefied fuels can lead to reduced pollutant emissions and improved burning rates, relative to untreated biomass, coal, and peat, but concurrently with the increased upstream emissions, energy consumption, and cost.^20−22^

The design and operation of a stove also impact the emission factors. Since, for a given appliance, absolute emissions are proportional to the quantity of fuel consumed, emissions from residential stoves can be reduced by increasing the thermal efficiency (TEs) of the appliance, as well as by improving its combustion characteristics. This twin-track approach is embedded in the EU Ecodesign Directive,^23^ which sets requirements for both the efficiency and emissions from residential, solid-fuel appliances.

A number of previous studies have looked in detail at the emissions from wood- and coal-fueled appliances.^17,24,25^ However, a systematic investigation into the emission behavior of organic particulate matter and gaseous species from fossil fuels, wood, and torrefied biomass combustion in domestic stoves of different designs using primary or secondary air supply has been rarely conducted. The novelty of this study derives from the measurement of particulate and gaseous emissions over the complete combustion cycle (including the critical, cold-start phase), for a range of fossil-based and bio-based fuels, and using a variety of measurement methods. In continuation of our previous work,^26^ the objectives of this study are (1) to compare the particulate matter emission factors obtained from measurements using the hot-filter method with those obtained using an aerosol chemical speciation monitor, and (2) to investigate the impact on stove thermal efficiency of burning a range of different biomass-, fossil-based, or pretreated fuels. Wood logs, torrefied olive stone (TOS) briquettes, smoky coal, smokeless coal briquettes, and peat were tested for comparison in two domestic multifuel stoves of different designs.

## 2 Materials and Methods

### 2.1 Stoves

**Figure 1:**
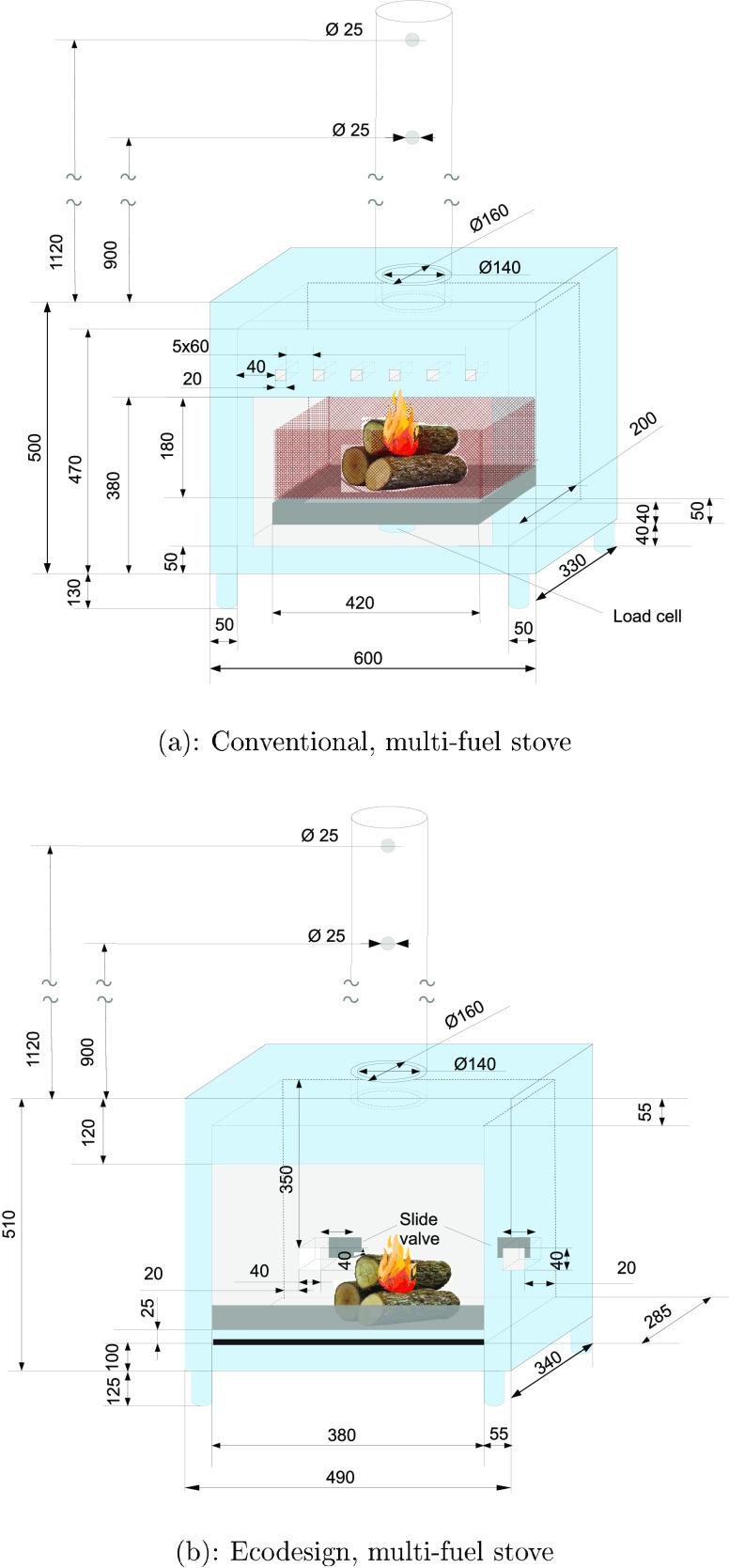
Domestic stoves at University College Dublin.

The burning experiments were set up in two stoves at University College Dublin (UCD), heretofore referred to as conventional and Ecodesign stoves. The primary differences between the stoves are their methods of control of air supply and their thermal rating. Figure 1a shows the conventional, multifuel stove, which has a nominal heat output of 11 kW, and has been described by Smith et al.^27^ The internal dimensions of the combustion chamber are 40 cm × 50 cm × 30 cm. A deflector plate lies across the top of the combustion chamber.

Primary combustion air enters through an inlet below the door of the stove; secondary air can be admitted through a series of holes above the door. Figure 1b shows a Waterford Stanley prototype multifuel stove, designed to comply with Ecodesign requirements, and with a nominal output of 9 kW. Primary and secondary air are drawn in through two valves on the rear side of the stove. An uninsulated chimney, with an inner diameter of 15 cm and length 110 cm, was attached to each stove outlet.

### 2.2 Experimental Procedure

For each combustion experiment, ≈3.5 kg of solid fuel and 100 g of solid firelighter (TESCO, Ireland) were placed in the stove. The test started when firelighters were lit, so most data streams—including the “hot-filter” PM measurements—incorporate the ignition and startup phase. However, PM data obtained using the ACSM and aethalometer methods generally began after the firelighters had burned out (approximately 15 min after ignition), to prevent AE33 overload and/or blockage of the dilution system. The duration of combustion tests varied from about 2 to 4 h. After each test, any solid residue remaining in the basket (conventional stove) or on the grate (Ecodesign stove) was classified as unburned fuel, weighed, and removed for elemental analysis. Small particles that fell through the basket (standard stove) or grate (Ecodesign stove) were collected, weighed, and classified as ash in further calculations. The experimental matrix for this study is shown in Table 1. Each experiment was conducted at least twice to check reproducibility.

**Table 1:** Experimental Conditions Used in the Present Study

	conventional stove		ecodesign stove	
fuel	primary air	primary + secondary air	primary air	primary + secondary air
wood logs	X	X		
TOS briquettes	X		X	X
peat	X			
ecobrite briquettes	X		X	X
smoky coal	X		X	X
firelighter	X	X		

A variety of methods was used to estimate PM emission factors for the firelighters alone. Using the ACSM + AE33 method, one test was conducted by burning 100 g of firelighter in an empty stove. A further three tests employed the ACSM + AE33 method during the ignition and startup phase of a standard-stove test using TOS briquettes. All emissions during the ignition and startup phase of these tests were attributed to firelighters. A separate series of tests used the hot-filter method to determine firelighter PM emissions, again using the standard stove. In these tests, ≈100 g of firelighter was placed in a bed of inert blocks, intended to simulate the presence of fuel blocks. Three such tests were performed using primary air only, and a further four tests incorporated secondary air.

### 2.3 Instruments

Samples of combustion products were extracted from three ports in the chimney. The lowest port, located 90 cm above the stove, supplied a Testo 350XL gas analyzer (TESTO, U.K.) that measured concentrations of O_2_, CO, CO_2_, and NOx in the raw exhaust. The second and third ports, both located 112 cm above the stove, supplied exhaust gas to the two separate PM sampling systems described below.

The hot-filter PM emission measurements were obtained by drawing a sample of the hot, raw flue gas through a 90 mm glass fiber filter (APFC09050, Merck Millipore, Ireland), which was supported on a circular, stainless steel mesh. The filter and mesh were in a housing that was heated to a nominal temperature of 120 °C, although control of this temperature was imperfect. A sample mass flow rate of 3.5 g min^–1^ was maintained using a Red-y Smart mass flow controller (Vogtlin Instruments, Switzerland). Filters used in the hot-filter sampling train were conditioned before and after sampling, by drying them in an oven at 160 °C for 2 h prior to being stored in a desiccator. After holding in a desiccator for 12 h, the filters were weighed on a College 150 weighing scale (Mettler Toledo, U.K.). The change in mass of the filter paper before and after the test is assumed equal to the mass of PM collected.

The second system measured PM emissions following cooling and dilution of the raw exhaust sample. This method attempts to simulate the household mixing of exhaust gases with ambient air, following their exit from the flue. PM mass measured using this approach is generally higher than obtained with hot-filter measurements because cooling of the sample encourages condensation of volatile organic compounds (VOCs) onto the surface of existing solid particles. The exhaust sample was first drawn through a PM_2.5_ cyclone and moisture trap, located approximately 2 m downstream of the sampling port. The sample then entered a diluter (DI-1000; Dekati Ltd), with a dilution range of 70–200:1, where it was diluted with compressed clean air. The cooled, diluted sample was then split and fed into an ACSM (Aerodyne Research Inc.) and an aethalometer (AE33, Magee Scientific).

The ACSM measured the nonrefractory, submicron aerosol (NR-PM_1_) composition (i.e., organic aerosol, sulfate, nitrate, ammonium, and chloride) with a time resolution of 2 min. The operating principle of the ACSM is described in Ng et al.^28^ and in Lin et al.^29^ Briefly, a Nafion dryer was used to dry the sample (flow rate 3 L min^–1^) before it entered the ACSM. Within the ACSM, the dried particles are focused into a narrow aerosol beam, which is directed onto a hot tungsten oven (≈600 °C) under a high vacuum. At 600 °C, the NR-PM_1_ components were vaporized, and the vaporized molecules were ionized by electron impact (70 eV). The resulting ions were analyzed by a quadrupole mass spectrometer. The mass concentration of NR-PM1 components was determined using ACSM software v1.6.1.0. Note that black carbon and other refractory components are not analyzed by the ACSM as they are not efficiently vaporized at 600 °C.

An aethalometer (AE33, Magee Scientific) was used to measure the black carbon (BC) from the same isokinetic sampling line as the ACSM, at a flow rate of 5 L min^–1^. A detailed description of the operating principles of AE33 is available in Drinovec et al.^30^ Briefly, the light absorption of the particles collected on the filter was measured at seven wavelengths (370, 470, 520, 590, 660, 880, and 950 nm) with a time resolution of 1 min.^34^ The change in optical attenuation at 880 nm was used to calculate the BC mass concentration using the mass absorption cross section of 7.77 m^2^ g^–1^.

For tests using the conventional stove, the fuel consumption rate was determined in real time using a load cell. The existing grate was removed from the stove, and fuel was placed instead in a specially designed basket, supported on the load cell as shown in Figure 1a. A tray, positioned below the basket, collected ash produced during combustion. For tests using only primary air, the inlet airflow rate was measured by installing a circular duct with an inner diameter of 5 cm connected to the primary air inlet. A pitot tube was positioned in this duct and connected to a differential pressure transducer (Control 699, Huber, Germany) to measure the flow rate of air into the stove.

In contrast to this arrangement, the Ecodesign wood stove was mounted directly on a weighing scale (Kern, Germany) with a precision of 0.005 kg, as shown in Figure 1b. The weight of the stove plus fuel was recorded manually once per minute, and burning rates were calculated from mass loss over time. The connection between the flue and the stove was modified to ensure that it did not interfere with weight measurements.

Additional sensors measured the flue gas temperature at the base and top of the flue, ambient temperature and pressure, and the temperature of and pressure drop across the PM filter housing. Data from all sensors was acquired and stored using LabVIEW VI software, which also presented a graphical and numerical display of key parameters on a PC monitor. All parameters were averaged over 10 s.

### 2.4 Emission Factor Calculation

Regardless of the measurement method employed, the PM emission factor is calculated as follows^31^
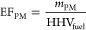
1For hot-filter measurements, the mass of PM collected on the filter is known. This is scaled up to the total flue emission using the ratio of total flue gas flow to sample flow. The sample flow rate is fixed at 3.5 g min^–1^; the flue gas flow is obtained by adding the measured inlet airflow to the measured fuel mass consumed during the sampling period
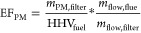
2Measurements obtained using the ACSM and AE33 are reported as mass concentrations (μg m^–3^) in the diluted exhaust sample and shown as *c*_meas_ in eq 3. This is converted to mass concentration (*c*_PM_) in the raw exhaust using the dilution ratio (DR) of the sampling process, which varies from test to test (and sometimes during a test). The dilution ratio (DR) for each test is obtained by comparing the CO concentration in the raw and the diluted exhaust gas.
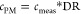
3Once the PM concentration (*c*_PM_) in the raw exhaust is known, it is multiplied by the total volume of flue gas emitted (*V*_flue_) during the sampling period. That volume is obtained from the measured mass of air and fuel consumed during the sampling period and an assumed density of 1.2 kg m^–3^ for the exhaust gas at standard temperature and pressure.
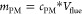
4Hence
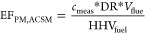
5Inlet airflow was measured directly for tests using the standard stove. This data was not available for the Ecodesign stove and was therefore estimated by assuming that the airflow through each stove was proportional to its nominal rating (9 kW for the Ecodesign stove vs 11 kW for the conventional stove). The CO_2_ and CO emission factors were calculated from their measured concentration in the flue gas and the mass flow rate of gas in the flue.

### 2.5 Thermal Efficiency Calculation

The main characteristics that are typically tested in the laboratory are safety, durability, and physical performance characteristics such as combustion quality, emissions, heat transfer, power range, and thermal efficiency.^32^ In the present study, thermal efficiencies (TEs) were calculated using eqs 6 and 7.^33^
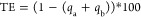
6In eq 6, *q*_a_ is the proportion of losses through specific heat in the flue gases, relative to the calorific value of the test fuel (as fired-basis), and *q*_b_ is the proportion of heat losses through combustible constituents in the residues, relative to the calorific value of the test fuel (as fired-basis). The total heat output (*P*) is calculated in eq 7(33)
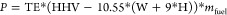
7In eq 7, HHV is the higher heating value, W and H are weight % of moisture and hydrogen in the fuel, respectively, and *m*_fuel_ is the mass of the tested feedstock. For this set of calculations, any unburned material still present on the stove grate was discounted, as in household operation; this material is retained and burned in any subsequent fires. Only material passing through the grate into the ash-pan was removed, with corresponding carbon/sulfur contents analyzed, as reported in the Supporting Information (Table S3). This modification accounts for the large fraction (>25%) of unburned material remaining on the grate after tests with smoky coal and Ecobrite.

### 2.6 Original Feedstock Characterization

Prior to chemical analysis, all fuels were milled in a laboratory-scale pulverizing mill LM1-P (LABTECHNICS, Australia) and sieved to <0.18 mm particle size. The elemental analysis of test fuels and solid residues was performed on an Analyser Series II (Perkin Elmer), according to the procedure described in ASTM D5373-02. Acetanilide was used as a reference standard, and the oxygen content was calculated by difference. Proximate analysis was conducted to determine the fraction of moisture, ash, volatiles, and fixed carbon according to the procedures described in ASTM D2216-19, ASTM D1102-84, ASTM D3175-11, and ASTM D3172-13. The higher heating value (HHV) was determined by a bomb calorimeter (IKA C-200) following ASTM D2015-95. Ash compositional analysis was performed by inductively coupled plasma-optical emission spectrometry (ICP-OES) with prior microwave digestion according to ASTM D6349-13. The Cl and S contents in the ash were analyzed by inductively coupled plasma-optical emission spectrometry/ion chromatography (ICP-OES/IC) at Celignis (Limerick, Ireland). Ash samples were dissolved in ultrapure water at 120 °C for 1 h, with the solution then filtered and analyzed by ICP-OES/IC.^34^

## 3 Results

### 3.1 Original Feedstock Characterization

Five fuels were tested in this study: torrefied olive stone (TOS) briquettes; manufactured, smokeless, coal ovoids (“Ecobrite”); sod peat; wood logs; and bituminous coal. The TOS briquettes and Ecobrite ovoids were manufactured at Arigna Fuels (Carrick on Shannon, Ireland).^21^ Ecobrite briquettes are produced by crushing anthracite to particle size < 3 mm, mixing with a 4.0% w/w starch binder, and pressed to a regular shape through a roll press. To produce TOS briquettes, olive stones are sieved to 1–3 mm particle size, torrefied at 280 °C as previously reported,^22^ and crushed.

The TOS powder is then mixed with a binder and pressed into a shape similar to that of coal-based briquettes. Peat sod was locally obtained from Leitrim, Ireland. The peat sod was cut in 11 cm logs and naturally dried prior to burning experiments. Wood logs cut from softwood grown in Ireland and bituminous coal from Silesia, Poland, were purchased from retail outlets.

Table 2 shows that bituminous coal and Ecobrite briquettes are high in carbon, sulfur, and ash compared to the biomass-based fuels. The high carbon (and correspondingly reduced oxygen) content in the fossil-based fuels, coupled with their low moisture content, increases their HHV relative to TOS, wood, and peat, confirming previous results.^35^ Ash analysis reveals that peat ash is higher in phosphorous, magnesium, and calcium than both wood logs and olive stones.^36^ Ash from bituminous and Ecobrite coals is higher in iron, aluminum, sodium, and silicon contents than the biomass-based fuels, as reported by Koukouzas et al.^37^ Overall, Table 2 illustrates the differences in a composition of raw fuels and pretreated biomass that could impact the combustion cycle in a domestic stove.

**Table 2:** Proximate, Ultimate, and Ash Compositional Analyses Using Firelighter, Wood Logs, Raw Olive Stones, Briquettes from Torrefied Olive Stones, Peat, Smokeless Briquettes from Coal (Ecobrite), and Smoky Coal Milled to 0.18–0.425 mm

properties	wood logs	raw olive stones	TOS briquettes	peat	Ecobrite	smoky coal	firelighter
Proximate Analysis/DIN EN 14775							
moisture (wt % as received)	15.7	15.5	9.4	26.5	6.3	1.3	0
ash at 550/815 °C, (wt % db)	0.2	0.8	2.1	2.1	3.9	4.9	1.7
volatiles (wt % db)	80.8	76	45.7	63.7	15.3	32.4	94.3
HHV (MJ kg^–1^ ar)/ISO 1928	19.2	22.2	24.3	19.8	32.8	31.3	35.9
LHV (MJ kg^–1^ ar)/ISO 1928	17.1	18.8	22.9	18.1	31.9	30.3	33.3
Ultimate Analysis (wt %, dry basis)/DIN EN 14775							
C	51.8	44.8	61.8	54.9	81.8	77.4	74.5
H	6.8	5.8	4.1	3.7	3.1	4.2	12.1
N	1.1	0.2	0.7	1.6	2.7	1.4	4.8
O	40.1	48.3	31.3	37.6	18.5	12.1	8.1
S	0.01	0.1	0.06	0.4	1.9	0.6	0.2
Cl	0.01	0.01	0.01	0.03	0.01	0.05	0.03
Ash Compositional Analysis in Feedstock (mg kg^–1^, dry basis)/DIN EN 15290							
Al	15	100	250	1550	9100	5900	
Ca	550	1650	1500	4000	3200	4600	
Fe	100	70	250	3600	6800	5600	
K	350	1600	1900	270	2300	550	
Mg	100	150	200	5000	450	1550	
Na	60	300	650	780	2100	2000	
P	70	100	150	1200	750	1500	
Si	90	1800	2000	6000	37 000	11 000	
Ti	1	10	20	90	600	260	

### 3.2 Changes in Fuel Mass

**Figure 2:**
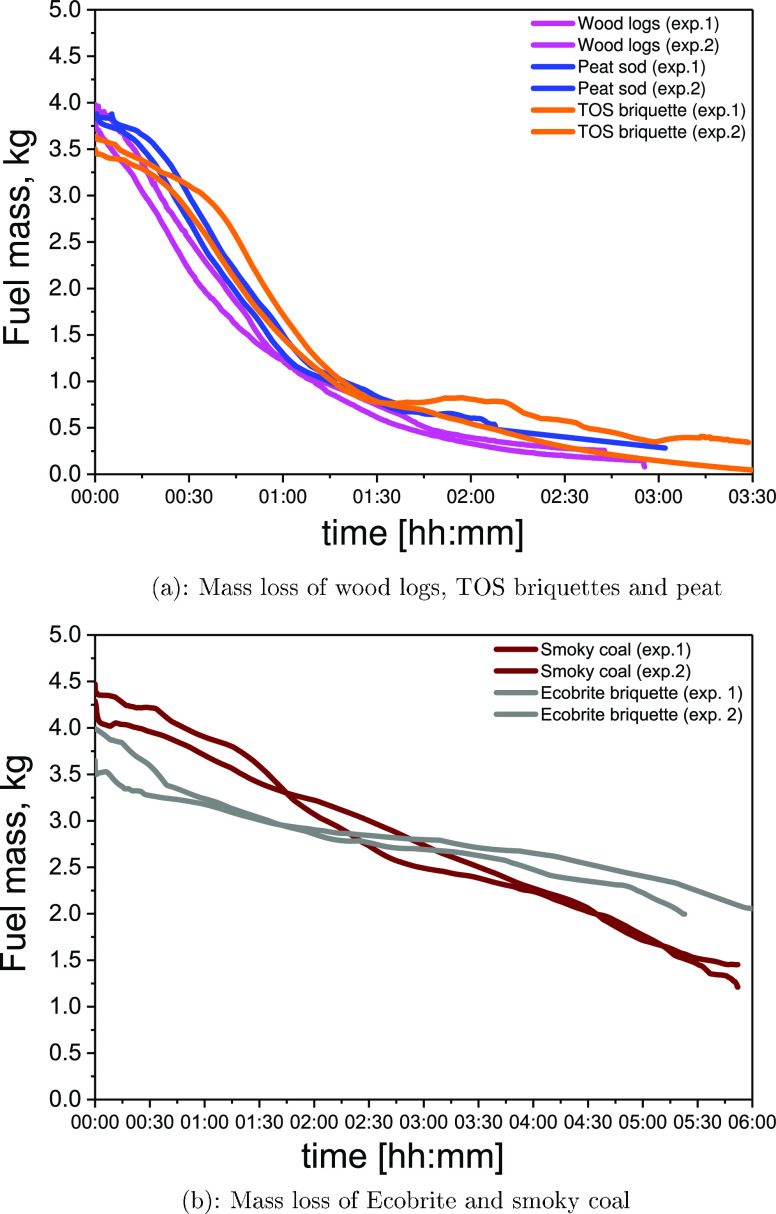
Mass changes in kilogram during combustion using primary air settings in the conventional stove.

**Figure 3:**
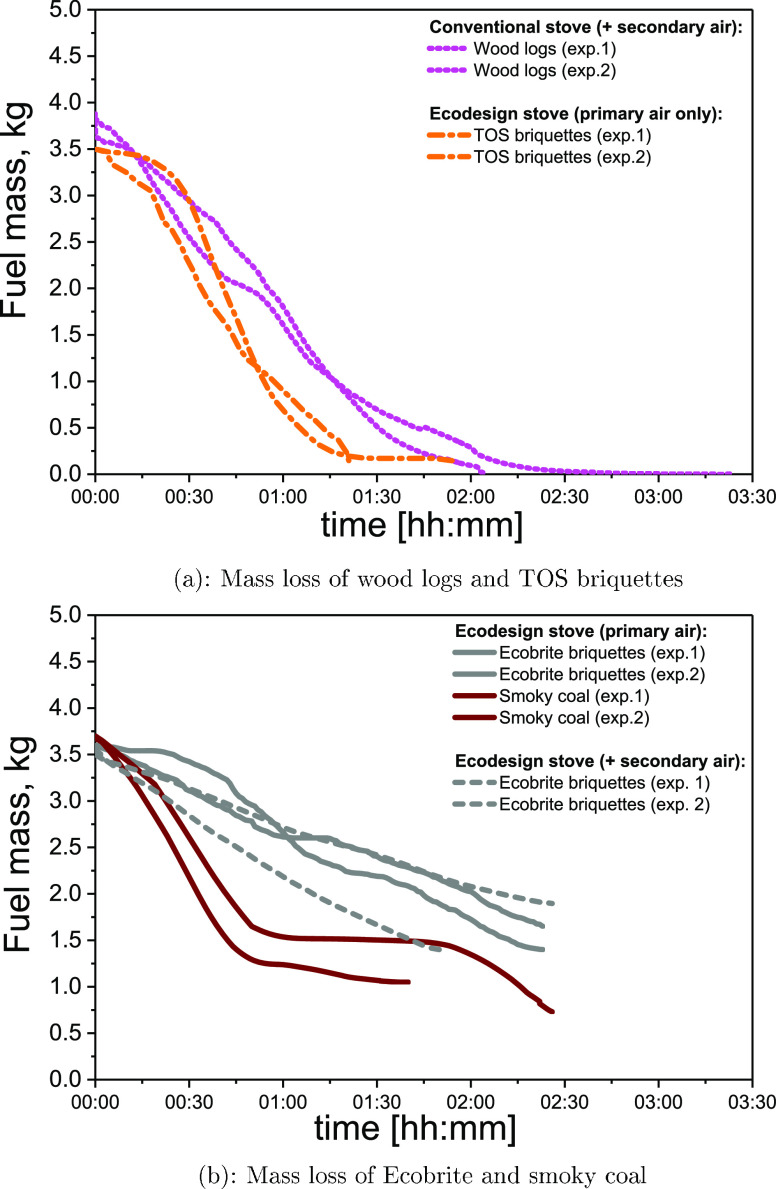
Mass changes in kilogram during combustion using primary only or primary combined with secondary air settings in the conventional or Ecodesign stove.

Figures 2 and 3 show the fuel consumption (mass loss) rates over time for tests in the conventional and Ecodesign stoves, respectively. Differences are observed among fuels, stoves, and air supply strategies. Generally, fuels with a high volatile content, i.e., wood logs, TOS briquettes, and peat, burned faster than Ecobrite briquettes or bituminous coal. Ecobrite briquettes, which have the lowest volatile content and highest fixed carbon, exhibited the lowest burning rate. Figure 2b shows that bituminous smoky coal burned less consistently than the other fuel types, possibly due to the nonuniform size and shape of lumps of coal, as suggested elsewhere.^38^ The regular shape and homogeneity of TOS and Ecobrite briquettes encouraged a more consistent burn. Coal type is characterized by relative size when screened, and these are generally of the type (from the largest to the smallest) trebles > singles > trebles > slack (or dross).^39^ There is also the potential for inconsistency depending on the part of the seam where the coal was mined or if the coal was blended in any way post mining. Ecobrite briquettes are manufactured by crushing to particle size < 3 mm, which are then bound together with a known quantity of binder and pressed to a regular shape through a roll press to improve durability. The coal briquettes have regular spacings when placed in the fire and allowed for air passage.

The high volatile contents of wood logs, TOS briquettes, and peat mean that these products generate combustible gases at relatively low temperatures, which promotes fast burning in stoves. The regular shape and homogeneity of torrefied biomass briquettes ensure a more consistent burn. Wood logs and peat sod were cut in larger pieces compared to briquettes, which might lead to differences in fuel stacking during the experiments, leading to air passages in both stoves and higher burn rates.

In both stoves, the combustion rate of wood logs and Ecobrite briquettes was quite insensitive to the use or omission of secondary air, as shown in Figure 3. All three of the fuels tested in both stoves burned more rapidly in the Ecodesign stove, indicating that stove design can significantly impact combustion.

In general, Figures 2 and 3 show that for a given fuel, stove, and air configuration, there is a difference between experiments 1 and 2. These results indicated that it is naturally expected to observe differences in any standard test procedure because each experiment is significantly affected by the performance of an individual stove user. Even if the duration of the test is in accordance with the standard test procedure,^27^ differences in mass loss can be observed due to the various distributions of coal pieces in a stove basket. This requires careful reconsideration of the existing standard procedures for solid-fuel burning. The standard procedures for monitoring of PM emission factors vary among different countries. Therefore, the significance of the present study relies on the use of several methodologies for PM emission monitoring, as discussed in Section 3.3.

### 3.3 PM Emission Factors

**Figure 4:**
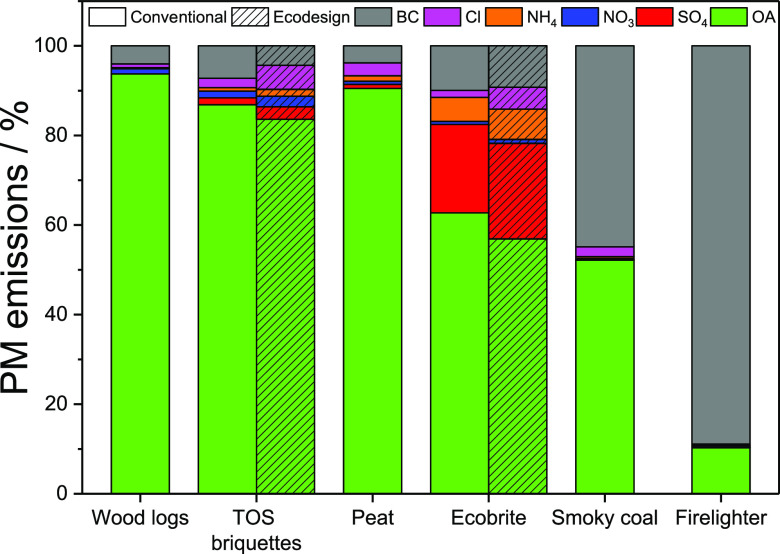
Relative composition of PM emissions measured using ACSM + AE33 from combustion of wood logs, torrefied olive stone briquettes, peat, Ecobrite briquettes, smoky coal, and firelighter in conventional and Ecodesign stoves using primary air supply shown as a percentage.

Figure 4 shows the relative composition of PM present in the cool, diluted flue gas, for each of the five fuels and for firelighters, as determined using ACSM and AE33.

For all five of the fuels tested, the chemical composition of PM was dominated by organic aerosol (OA), which accounted for between 52 and 93% of the PM mass. Black carbon (BC) constituted less than 10% of PM mass for all fuels except for bituminous coal, where it accounted for almost 45%. Sulfate (SO_4_) accounted for ≈20% of the PM obtained from Ecobrite smokeless coal, reflecting the higher sulfur content of the raw fuel. Other inorganic species—nitrate, ammonium, and chloride—represented only very minor fractions of PM mass in all cases. It is also notable that PM composition was not influenced significantly by stove design although, as discussed below, stove design does influence the total mass of PM emitted. The composition of PM emissions from TOS briquettes and Ecobrite showed only small variations between the conventional and Ecodesign stoves. PM from TOS briquettes showed a higher concentration of SO_4_ (2.8%) in the Ecodesign stove than in the conventional stove (1.5%). For Ecobrite briquettes, the Cl concentration was higher in PM from the Ecodesign stove (4.9%) than from the conventional stove (1.5%). These differences might be explained by the better air recirculation during burning of biomass and smokeless briquettes in the Ecodesign stove, leading to the more extensive release of chlorides and sulfates.

For most of these fuels, the mass of OA in PM is closely linked to the volatile content of the raw fuel: a higher volatile fraction leads to higher OA emission. TOS briquettes are an exception to this rule—despite a moderately high volatile fraction, OA emissions are low. This is probably because torrefaction removes a multitude of products including water, tars, and a great many degradation products from the lignocellulosic structure.^40^ Depending on the torrefaction processing conditions, the composition of emitted products changes markedly from relatively simple oxygen-containing polar compounds at temperatures of 220–260 °C (e.g., acetic acid, furfural, and methoxyphenols) to more complex and higher-molecular-weight tars that are cross-linked sufficiently to form viscous hydrophobic and predominantly hydrocarbon-based compounds (macro-aromatic structures) when the reaction temperature is raised above the autothermal temperature, which is generally in the range of 270–300 °C.^41^ In contrast to solid fuels, the composition of PM from firelighters was dominated by BC (88.9%). OA accounted for only 10.3% of the firelighter PM, with minor traces of inorganic species again present (see the Supporting Information Table S1 for numerical data). The measurement of high BC concentration in PM from the firelighter affected the design of experiments and data processing. Thus, the impact of measurement method on PM emission factors was investigated by presenting the results with “including ignition phase” and “excluding ignition phase”.

### 3.4 Effect of Measurement Method on PM Emission Factors

**Figure 5:**
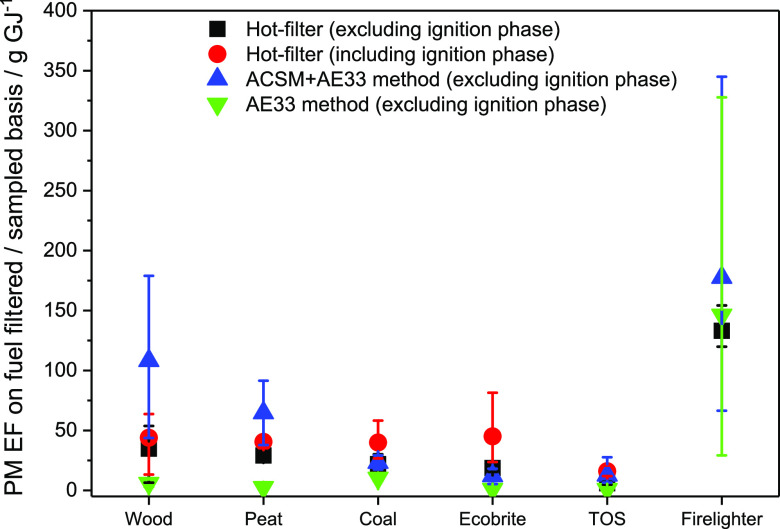
PM emission factors measured gravimetrically using the hot-filter system including and excluding the ignition phase, ACSM + AE33, and only AE33 excluding the ignition phase from combustion of wood logs, torrefied olive stone briquettes, peat, Ecobrite briquettes, smoky coal, and firelighter in a conventional stove with primary air supply shown in g GJ^–1^.

As previously noted, PM emissions in this study were measured using two different methodologies. A combination of ACSM plus AE33 aethalometer was used to measure the PM concentration in cool, diluted flue gas, whereas a hot-filter method measured the PM concentration in the hot, raw flue gas. Two versions of the hot-filter PM EF are presented in Figure 5: including ignition phase and excluding ignition phase.

The including ignition phase data attributes all PM emissions to the test fuel. In reality, however, some of this PM derives from the firelighters. The excluding ignition phase data estimates the firelighter contribution using our measured firelighter EF and the mass of firelighter used in each test. This estimated firelighter contribution is then subtracted from the total PM emissions before calculating the excluding ignition phase EF.

Figure 5 presents the PM EF for each of the five fuels, and for firelighters, obtained using each approach. All data in the figure pertain to tests with the standard stove, using primary air only. The results showed that the PM emission factor determined for a given fuel depends on the measurement method employed. For wood and peat, the PM EFs determined using cool, diluted exhaust are substantially higher than those determined using the hot-filter method. This correlates well with the high proportion of OA in the PM from these fuels and implies that a significant fraction of the OA emissions from these fuels can remain in the vapor form at temperatures around 120 °C. These vapors cannot be trapped by a filter and therefore do not contribute to the PM emissions measured using the hot-filter approach. In the cooled, diluted flue gas, however, many volatile organic compounds (VOCs) will tend to condense from vapor to liquid as cooling proceeds. Solid particles act as condensation nuclei for these VOCs, which are adsorbed onto the particle surface. This increases the mass of PM present in the cooled exhaust, which is measured using the ACSM + AE33. For fuels with a lower volatile content, such as coal and Ecobrite, the effect of VOC condensation is reduced, and the PM EF determined using a heated filter closely matches that obtained in the cooled, diluted exhaust.

Substantial variations in PM EF are observed for all fuels, irrespective of the measurement approach. Such variability is inherent in the combustion of solid fuels in a domestic stove. It can be attributed to differences in the size, shape, moisture content, and volatility of the fuel elements used from test to test and/or to differences in the physical arrangement of the fuel elements and firelighters in the stove prior to ignition. To be representative, a PM emission factor should therefore be derived from a suitably large number of repeat tests.

Firelighters have a very high PM emission factor, regardless of the measurement method employed. Inclusion or omission of these emissions can make a substantial difference to the PM EF calculated for a particular fuel. Qualitatively, emission factors determined using the ACSM and AE33 were broadly comparable to those obtained using the hot-filter approach, as seen in Figure 5. In particular, the PM emission factor for firelighters is found to be much higher than for any of the fuels. Quantitatively, PM emission factors determined from gravimetric measurements are lower than those derived from ACSM and AE33 measurements, for most fuels. This is primarily because the hot-filter method does not capture light condensable organic compounds (COCs). The impact of the measurement method on PM emission factors was further investigated in a domestic stove of different designs, as discussed in Section 3.5.

### 3.5 Effect of Stove Design on PM Emission Factors

**Figure 6:**
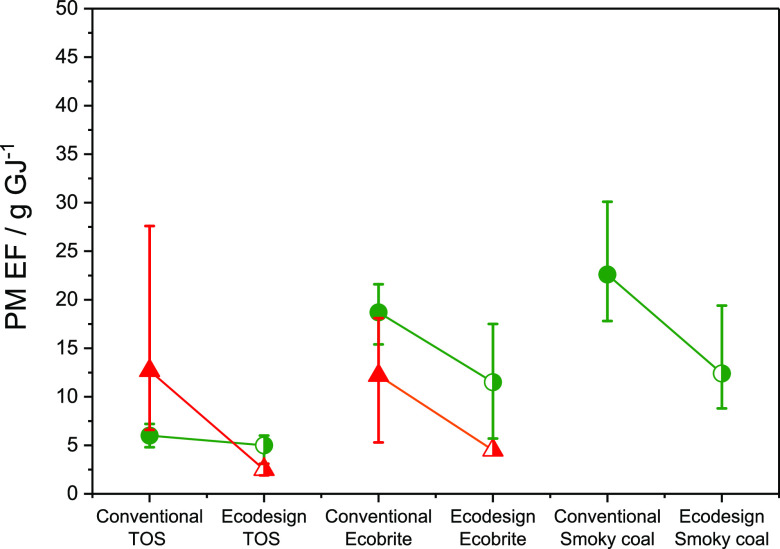
PM emission factors from combustion of smoky coal, torrefied olive stones, and Ecobrite briquettes in the conventional or Ecodesign stove using primary air supply. Triangles denote EF obtained using ACSM + AE33; circles denote EF obtained using the hot-filter approach.

Figure 6 illustrates the impact of stove design on PM emissions, for three of the fuels tested. It is clear that switching from the standard stove to an Ecodesign stove reduced the PM EF significantly for all three fuels. However, it is also notable when burning TOS briquettes that the Ecodesign stove reduces PM EFs by over 80% when based on measurements in the cooled, diluted exhaust but by less than 20% when using the hot-filter method. This may indicate that a primary benefit of using the Ecodesign stove when burning TOS briquettes is a substantial reduction in the mass of OA that leaves the combustion chamber.

Another benefit of the Ecodesign stove application is a simple control of the additional air supply that increases the fuel oxidation and reduces formation of emissions, as discussed in Section 3.6.

### 3.6 Effect of Secondary Air Supply on PM Emission Factors

**Figure 7:**
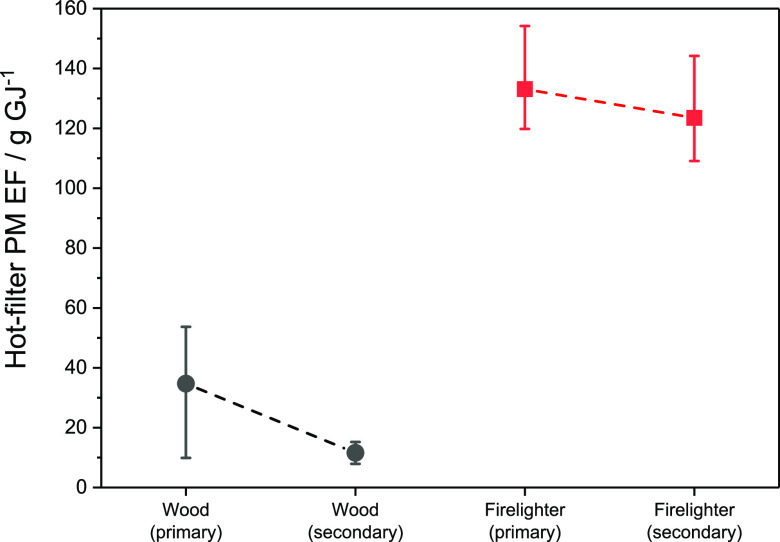
PM emission factors from combustion of wood logs and firelighter in the conventional stove using primary air supply or secondary air supply.

Figure 7 presents the PM EFs obtained with the conventional stove, burning wood logs, or firelighters, based on the hot-filter method.

These results indicate that the use of secondary air can reduce PM from wood logs by about two-thirds due to the improved mixing of fresh air with preliminary combustion products. Operator behavior can clearly exert a strong, adverse influence on PM emissions when burning solid fuels. On the other hand, no significant reduction in PM EF is observed for the firelighters. This is because substantial excess air is already available when firelighters alone are burned. It has already been noted (Section 3.3) that PM from firelighters is dominated by BC. The incorporation of cool secondary air is therefore unlikely to yield a substantial reduction in BC emissions. It is also evident that the PM emission factor for firelighters is substantially higher than for any of the fuels tested. Because firelighter PM is dominated by BC, whereas PM from most solid fuels is dominated by OA, kerosene-based firelighters accounted for between 78 and 97% of BC emitted from combustion of almost all solid fuels in this campaign. Bituminous coal was the only exception, with firelighters accounting for “only” one-third of BC emissions.

As previously stated, many standard test protocols for domestic heating appliances do not count particulates from the lighting-up phase because these standard tests light the fuels using a fixed mass of propane or butane gas. Emissions are measured only once the stove has reached a stable burning condition and therefore reflect the minimum likely level of emission. In the present work, however, kerosene-based firelighters displayed a PM emission factor 10 times higher than those of typical solid fuels. These firelighter emissions also overlap with potentially high levels of boil-off emission from cool fuel elements during the lighting-up phase, particularly for untreated biomass-based fuels such as wood and peat. PM emissions during the ignition and lighting-up phases are therefore very much higher than during the stable combustion phase. From an air-quality and human-health perspective, it is essential that these startup emissions are accounted for when regulating PM emissions from domestic appliances. The combustion of solid fuels is always accompanied by the release of gaseous species. Moreover, the calculation of thermal efficiency of domestic stoves includes the measurement of carbon dioxide. The concentrations of gaseous species are discussed in Section 3.7.

### 3.7 Gas Composition

**Figure 8:**
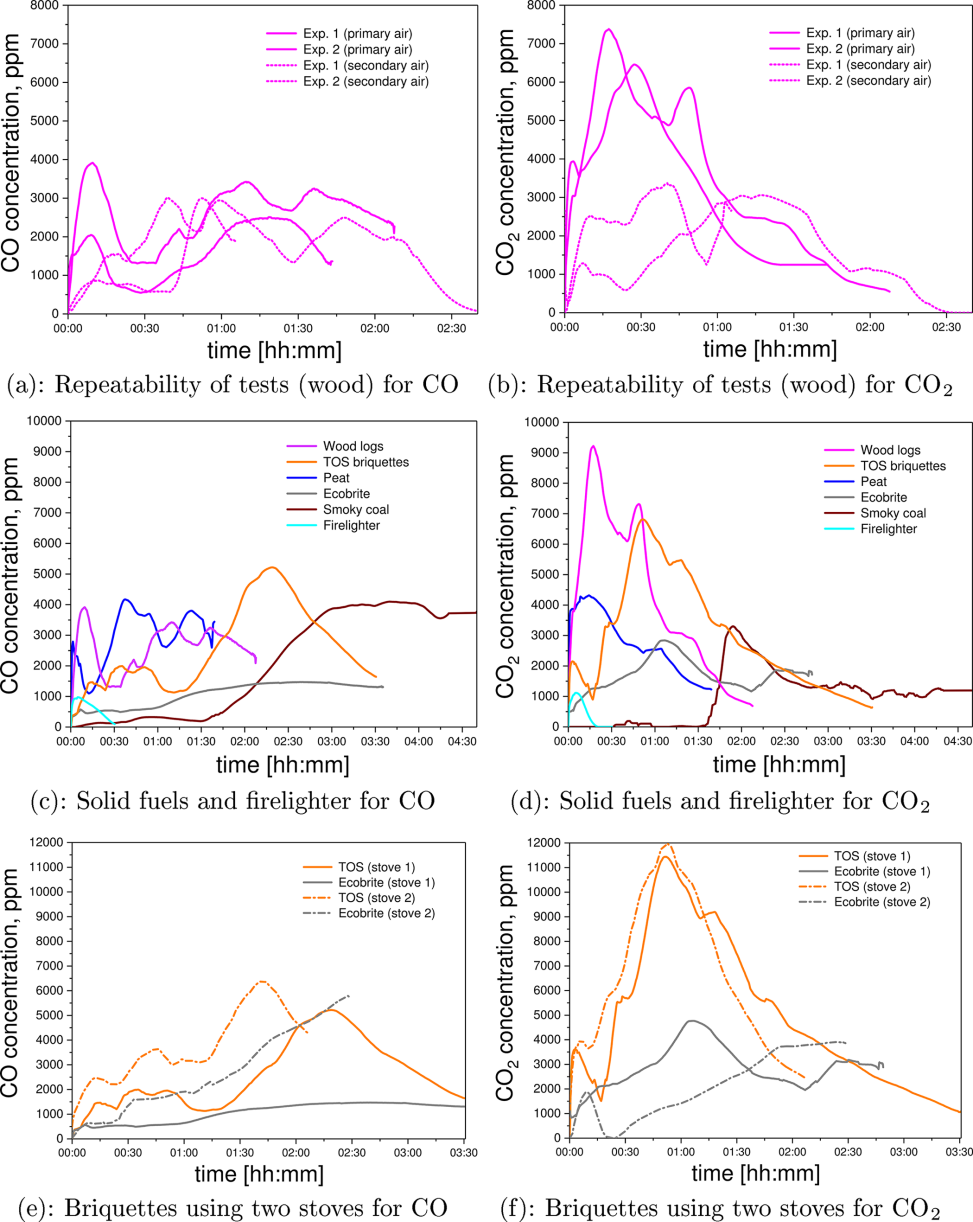
Gas composition (CO, ppm, and CO_2_ in %). (a, b) Test repeatability with wood logs in a conventional stove using primary air supply or with the addition of secondary air supply. (c, d) CO and CO_2_ compositions of burned wood logs, peat, smoky coal, firelighter, TOS, and Ecobrite briquettes using a conventional stove. (e, f) CO and CO_2_ compositions of burned TOS and Ecobrite briquettes using a conventional or an Ecodesign stove.

Figure 8 shows measured concentrations of CO_2_ and CO in the raw exhaust for each test. For tests using the conventional stove, peak CO levels were generally in the range of 3000–6000 ppm but showed a large dependence on the fuel type and flame phase, i.e., “intense” or “weak”. Large variations were observed between repeat runs, as shown in Figure 8a,b. It is also possible to distinguish some differences in the overall pattern of emission, between fossil-based fuels on the one hand and biomass-based fuels on the other, as shown in Figure 8c,d.

For fossil fuels, the CO concentration is relatively low during lighting-up/flaming combustion, starts to rise as the heating output decreases, and is at a maximum during the smoldering phase. Biomass-based fuels are much more variable in their CO output, with CO emission peaks likely to occur at any time during the test, confirming the previous results of Mitchell et al.^25^ Peak CO_2_ concentration is associated with intense flame periods, characterized by large flames in the stove. With the conventional stove, these peaks are observed 10–16 min after ignition for wood logs and peat, 1 h after ignition for TOS and Ecobrite briquettes, and 2 h after ignition for bituminous coal. As the flame intensity falls, “weak flame” periods lead to a decrease in the CO_2_ concentration coupled with lower combustion temperatures.^42^Figure 8e shows that changing to an Ecodesign stove did not affect the concentration of CO.

Moreover, the differences between solid fuels had a stronger influence on the gas composition than the stove type, as seen in the similar CO and CO_2_ concentrations for TOS briquettes burned in each stove, as shown in Figure 8e,f. Distribution of CO and CO_2_ was different for tests with primary air only and those with the addition of secondary air, as shown in Figure 8a. Primary air entering the stove directs air underneath the combustion zone, whereas the stoves have a secondary air setting that controls the rate of burn when using wood or high-volatile fuels and is also used to keep the glass clean. Therefore, experiments with only the primary air open showed more heterogeneous CO and CO_2_ gas release than the experiments with the addition of secondary air supply. This is partially due to the dilution factor with excess air.

### 3.8 Thermal Efficiency Factors

**Figure 9:**
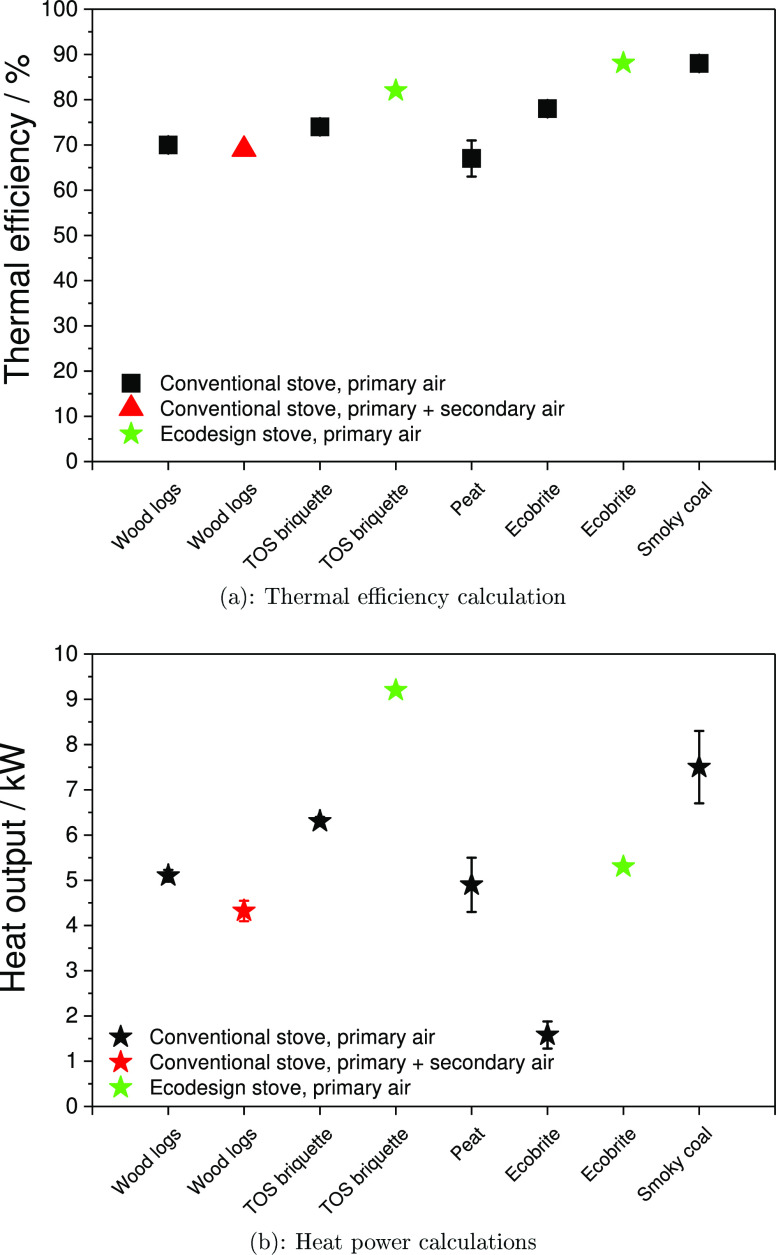
Calculated thermal efficiencies and heat power for wood logs, torrefied olive stone (TOS) briquettes, peat, Ecobrite, and smoky coal using primary air and with the addition of secondary air supply.

Thermal efficiencies (TEs) and heat power values were calculated using eqs 6 and 7.^43^ The values were calculated as an average of two experiments. As can be observed in Figure 9b and the Supporting Information (Tables S3 and S4), the thermal efficiency for Ecobrite was high (≈78%) and the relative heat output was low (1.7 kW). This was largely due to the large amount of unburned product retained on the grate and which, due to the high fixed carbon content of Ecobrite (≈75%), require stove combustion temperatures to be elevated to >800 °C for extended periods, which is curtailed when the stove is “slumbering”.

This elevated temperature ensures complete combustion of carbon to CO_2_, with a corresponding increased heat output. High thermal efficiencies are achieved when flue gas losses are curtailed. Flaming combustion of high-volatile materials (wood, TOS briquettes, and peat especially) creates large volumes of very hot gas, raising the average flue temperature over shorter average durations than fuels with high fixed carbon contents. This accounts for significant heat losses in the flue gas, lowering thermal efficiencies. A further factor is the increased flue gas airflow and raised flue temperature as a result of a rapidly burning fuel, which in turn draws more air into the inlet, which increases the combustion rate. Figure 9a suggests that the secondary air addition has very little effect on the stove efficiency. Secondary air addition to the wood log burning is a way to both cool the flue gas and increase the volume, thus increasing the stove efficiency. However, the secondary air addition in conventional stoves seems to have a compensating effect from the experimental environment (room temperature, room air circulation, occasional stove door opening, etc.) on the stove efficiency. Operator behavior can clearly exert a strong, adverse influence on PM emissions and also on the stove efficiency.

The burning of smoky coal and Ecobrite was strongly affected by the user-specific differences in a pile preparation prior to fuel ignition. During the entire experimental campaign, the user-specific features, i.e., height and shape of the fuel pile and placement of coal briquettes or lumps in a basket, had a significant influence on the burning rate. The increased thermal efficiency from smoky coal and Ecobrite is due to the largest proportion of heat emitted from radiation, leading to the glowing or reddening of coal. This provides sustained heat output for extended periods, long after flaming combustion has subsided, as shown in Figure 9b. This type of combustion is synonymous with low-smoke fuels and leads to a corresponding reduction in PM emissions.

The maximum heat output was achieved with TOS briquettes, which is a consequence of high volatile content (45.7%), low moisture content (<10%), and raised higher heating value (≈24.3 MJ kg^–1^), which compounded to a consistently fast burn, but with greater flue gas losses, as shown in the Supporting Information (Tables S3 and S4). Burnout was almost complete, which meant unburned losses were negligible. In general, the TEs clearly showed that a combination of fuel type (with differences in mineral matter, carbon/hydrogen content, moisture, and lower heating value), stove design, airflow settings, and user type plays an important role in the calculation of heat outputs. These important findings in combination with the current policy reports will be further discussed in Section 4.

## 4 Discussion

This study showed that wood logs generated the most amount of PM and CO_2_ emissions, whereas TOS briquettes and Ecobrite produced less PM emissions than other solid fuels, as shown in Section 3.3 and in the Supporting Information (Figure S5). The PM emission factors for solid fuels ranged from 0.2 to 108.2 g GJ^–1^ net depending on the stove type, air supply, and method of PM determination. In general, the literature reports a range of values for PM emission factors for wood, woodchips, and pellets made from triticale and miscanthus burning varying from 3 to 170 g GJ^–1^.^44−46^^44−46^ Thus, the present PM emission factors for wood log burning using both ACSM and gravimetric methods were in the range of previously calculated PM emission factors (34.8–108.2 g GJ^–1^).^42^ In the present study, Ecobrite and TOS briquettes generated the lowest PM emission factors (6.0–18.7 g GJ^–1^), lower than the PM emission factors reported (51.5–98.1 g GJ^–1^) for smokeless fuel in the literature.^23,47^ The Ricardo report estimates that the total annual mass of PM_2.5_ emissions from residential burning of smoky coal in Ireland is 2451 tons (31% of the total PM emissions), peat is 4858 tons (62% of the total PM emissions), and biomass is 588 tons (7% of the total PM emissions).^47^ However, uncertainty surrounds the reporting of biomass fuel consumption, which may be 50–200% higher, when nontraded wood is included.^6,48^

Burning of these products accounts for over 93% of the total residential particulate PM_2.5_ emissions for the whole of Ireland. If, as proposed, domestic combustion of smoky coal and peat were 100% substituted with unprocessed biomass fuels, our results suggest that this could lead to significant increases in particulate air pollution. As noted in Section 3.4, the absolute level of PM emissions determined for a particular test depends on the measurement method employed. Cooling and dilution of the flue gas prior to sampling ensure that condensable organic compounds (COCs) are included in the PM measurements and therefore tend to yield a higher PM EF than samples taken from the hot, raw flue gas. The measurement equipment required, however, is substantially more expensive, more delicate, and more cumbersome than the hot-filter system and requires significant technical expertise for setup and operation. The associated dilution system is prone to blockage (particularly during the PM-intensive ignition/lighting-up phases) and introduces significant uncertainty regarding the instantaneous dilution ratio, which is central to the calculation of PM emissions. Moreover, the literature suggests that PM EF is directly affected by the level of dilution employed.^49,50^ The hot-filter method, in contrast, is relatively simple and robust and captures PM from all stages of the combustion process, including the all-important ignition and lighting-off phases. However, it does not capture volatile organic matter that condenses at a temperature lower than that of the filter itself and may therefore underestimate PM emissions for fuels with a high volatile content.

Based on the data in Section 3.3, the average PM_2.5_ emissions arising from domestic solid-fuel combustion across the whole of Ireland in 2011 were 360 g GJ^–1^. Our results suggest that, if torrefied fuels were substituted for smoky coal, peat, and unprocessed wood fuels, the reduction in PM_2.5_ emissions would be in the range of 63%. This is supported by previous results describing the benefits of torrefaction pretreatment leading to reduced formation of PM emissions.^17^ The decrease in PM emissions caused by torrefaction is likely a culmination of different effects such as pretreatment, physical structure of briquettes, elemental composition, and reduction of moisture content, as previously reported.^21,25^

When compared with Ecobrite, smoky coal had similar values for elemental composition and calorific values, as shown in Table 2. However, smoky coal showed greater PM emission factors than the burning of smokeless coal generates. Thus, the results indicated that the combination of elemental composition and proximate analysis is a better indicator of the tendency of any fuel to generate particulate matter, rather than elemental composition alone.

Operator behavior (e.g., control of air supply, configuration of fuel and firelighters prior to ignition) plays a significant role in determining PM emissions and thermal efficiency during stove operation. Due to large differences in the fuel morphology, it is not always possible to follow guidelines given by stove manufacturers, which leads to measurement uncertainty. In addition, differences in principles of measurement methods, i.e., whether PM is measured in the hot, raw flue gas or in cooled, diluted flue gas, significantly influence the value calculated for PM emission factors. The present results using the OA method confirmed that all three fuels, i.e., smoky coal, peat, and biomass, can increase particulate air pollution. With regard to the stove type, the present results showed that the Ecodesign stove reduced PM emissions from burning of biomass and coal by 5–45%, in agreement with previous results.^10,51^ The TOS briquettes emitted the least amount of particulate in both stoves.

The addition of secondary combustion air in both stoves led to significant reductions in PM emissions (≈30–60%). A previous study reported that PM emissions can be reduced by up to 90% by installing energy-efficient fans/blowers in test stoves.^52^ The authors observed that total particle numbers remained unchanged but that particle growth was inhibited when secondary air was injected into the stove.^53^ A synergistic combination of factors such as biomass pretreatment, use of a modern stove type, and appropriate control of secondary air supply can reduce PM emissions from domestic solid-fuel combustion and must be considered during the design of new-generation stoves. The results presented here show that these factors also affect the heat output and stove efficiencies. However, the interpretation of interaction between these factors depends strongly on the use of standards for the calculation of PM emission factors and thermal efficiencies.

Introduction of the Ecodesign directive for solid-fuel heaters in 2022 should assist with reducing PM, NOx, and CO emissions over a number of years; however, significant emission reduction could be achieved sooner if consumers were encouraged to switch to less polluting solid fuels.

## 5 Conclusions

The novelty of the present work derives from the use of dual-measurement methods to determine PM emission factors from domestic stoves. These emission factors depend on user behavior, on stove-specific features, and on the type of measurement method used. Organic aerosols were the dominant constituents of PM emissions observed in our tests, regardless of the compositional differences between the fuels. However, black carbon constituted up to 90% of the PM emitted by firelighters, and firelighters also displayed a PM emission factor far higher than any of the fuels studied. These findings will be explored further in a forthcoming paper. This study also suggests that thermally pretreating biomass using torrefaction can significantly reduce emissions compared to wood logs, peat, and smoky coal. A countrywide switch to (1) Ecodesign-approved stoves and (2) lower-emitting solid fuels could have a significant impact on air pollution reduction in Ireland. However, individual users will continue to exert a substantial, uncontrollable influence on the absolute level of PM emission from manually controlled domestic stoves.

## Supporting Information Available

Emission factors (Section S1); gas composition (Section S2); CO and CO2 emission factors (Section S3); thermal efficiency (Section S4) (PDF)
