# Supplementary material for: Study of Emissions from Domestic Solid-Fuel Stove Combustion in Ireland
Source: Energy Fuels. 2021 Feb 26;35(6):4966–78. doi: 10.1021/acs.energyfuels.0c04148 (PMC8277100; doi:10.1021/acs.energyfuels.0c04148)
Supplement: Supplementary file 1 — ef0c04148_si_001.pdf [file ef0c04148_si_001.pdf]

A study of emissions from domestic solid fuel stove combustion in Ireland

Anna Trubetskaya,[*,†] Chunshui Lin,[‡,¶] Jurgita Ovadnevaite,[*,§,‖] Darius Ceburnis,[§,‖] Colin O'Dowd,[§,‖] JJ Leahy,[†] Rory F.D. Monaghan,[⊥,‖] Robert Johnson,[#] Peter Layden,[#] and William Smith[*,@]

†*Department of Chemical Sciences, University of Limerick, Limerick, Ireland*

‡*State Key Laboratory of Loess and Quaternary Geology, Key Laboratory of Aerosol Chemistry and Physics, Institute of Earth Environment, Chinese Academy of Sciences, Xi'an 710061, China*

¶*CAS Center for Excellence in Quaternary Science and Global Change, Chinese Academy of Sciences, Xi'an 710061, China*

§*School of Physics and Centre for Climate and Air Pollution Studies, Ryan Institute, National University of Ireland Galway, University Road, Galway, Ireland*

‖*MaREI, the SFI Research Centre for Energy, Climate and Marine, Galway, Ireland*

⊥*School of Engineering and Ryan Institute, National University of Ireland Galway, Galway, Ireland*

#*Arigna Fuels, Arigna Carrick-on-Shannon Co. Roscommon, Ireland*

@*Department of Mechanical Engineering, University College Dublin, Dublin, Ireland*

E-mail: anna.trubetskaya@ul.ie; jurgita.ovadnevaite@nuigalway.ie; william.smith@ucd.ie

S-1 Emission factors

Chemical composition of particulate matter from burning of wood logs, torrefied olive stones, peat, Ecobrite briquettes, smoky coal and firelighter is shown in Table S-1.

Table S-1: Chemical composition of particulate matter from burning of wood logs (WL), torrefied olive stones briquettes (TOSB), peat, Ecobrite briquettes (EB), smoky coal (SC) and firelighter (FL) measured by ACSM+AE33 in conventional and Ecodesign stoves and shown in %.

**Component**	**WL**	**TOSB**		**Peat**	**EB**		**SC**	**FL**
**Stove type**	1	1	2	1	1	2	1	1
**OA**	93.73	86.87	83.59	90.49	62.72	56.89	52.17	10.27
**Cl**	0.8	2.08	5.38	2.91	1.54	4.87	2.18	3.39
**NH_4**	0.24	0.75	1.52	1.21	5.34	6.77	0.41	0.1
**NO_3**	1.15	1.52	2.35	0.69	0.66	0.85	0.22	0.36
**SO_4**	0.03	1.52	2.81	0.93	19.63	21.36	0.15	0.03
**BC**	4.01	7.25	4.33	3.78	9.88	9.25	44.87	88.9

The PM emission factors were calculated from gravimetric measurements using the filtering system and ACSM and AE33 methods.

Table S-2: PM emission factors (g GJ^{-1}) measured using gravimetrically using the hot-filter system including and excluding ignition phase, ACSM+AE33 and only AE33 excluding ignition phase from combustion of wood logs, torrefied olive stones briquettes, peat, Ecobrite briquettes, smoky coal and firelighter in conventional stove with primary air supply. The results are shown as mean, minimal and maximal values.

	**WL**	**TOSB**	**Peat**	**EB**	**SC**	**FL**	**TOSB**	**EB**	**SC**	**WL**	**FL**
	**primary air**									**+ secondary air**	
	**Conventional**						**Ecodesign**			**Conventional**	
HF (excl. ign.), mean	34.8	6.0	29.1	18.7	21.9	133.1	5.0	11.5	11.5	11.6	123.5
HF (excl. ign.), max	53.7	7.2	33.0	21.6	30.1	154.3	6.0	17.5	18.6	15.2	144.2
HF (excl. ign.), min	6.4	4.8	25.3	15.4	16.9	119.9	3.1	5.7	7.9	7.9	109.1
HF (incl. ign.), mean	43.9	16	40.5	45.1	39.9	-					
HF (incl. ign.), max	63.7	20.8	45.7	81.4	58.2	-					
HF (incl. ign.), min	13.2	11.9	35.3	23.7	26.7	-					
ACSM, mean	108.2	12.7	64.7	12.2	23.3	177.6	4.8	3.2			
ACSM, max	179.0	27.6	91.6	18.1	29.2	327.7	6.7	4.1			
ACSM, min	43.7	6.6	37.9	5.4	17.4	66.5	2.9	2.3			
AE33, mean	6.0	0.9	2.5	1.0	10.3	146.2	0.2	0.3			
AE33, max	9.2	1.6	3.5	1.8	12.9	327.7	0.3	0.4			
AE33, min	2.8	0.3	1.4	0.5	7.7	29.2	0.1	0.2			

S-2 Gas composition

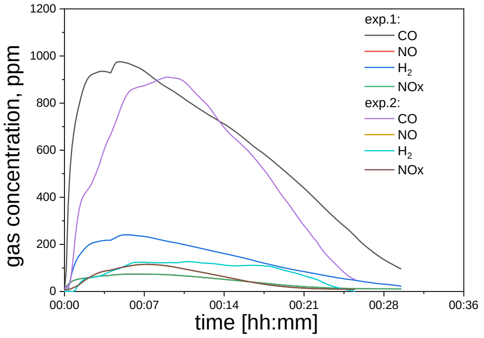

1(a): Firelighter (CO, NO, H_2, NOx)

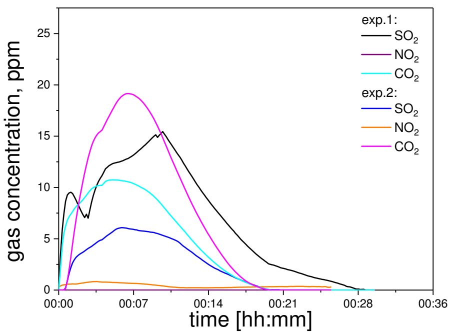

1(b): Firelighter (SO_2, NO_2, CO_2)

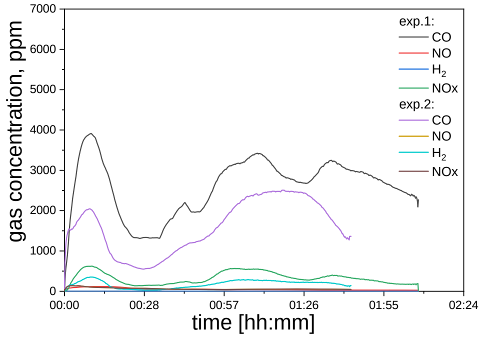

1(c): Wood logs (CO, NO, H_2, NOx)

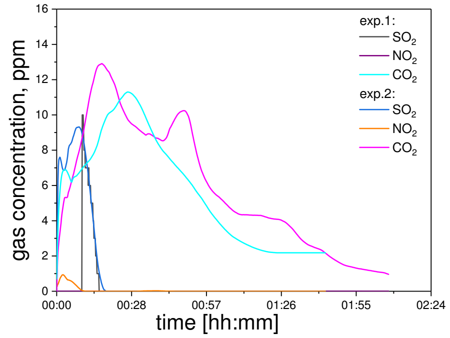

1(d): Wood logs (SO_2, NO_2, CO_2)

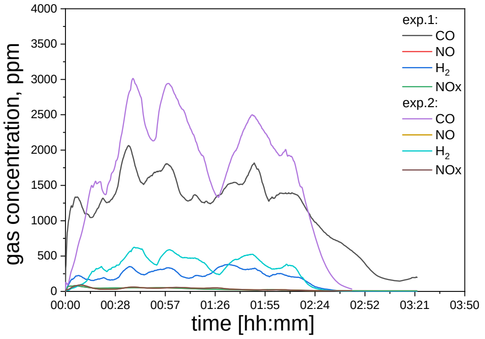

1(e): Wood logs with secondary air (CO, NO, H_2, NOx)

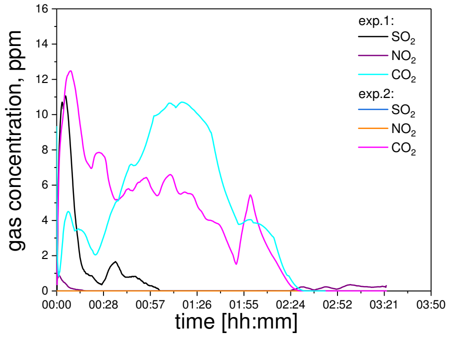

1(f): Wood logs with secondary air (SO_2, NO_2, CO_2)

Figure S-1: Gas composition (CO, NO, H_2, NOx, SO_2, NO_2, CO_2) during combustion of firelighter and wood logs using only primary air supply or with the addition of the secondary air in the conventional stove.

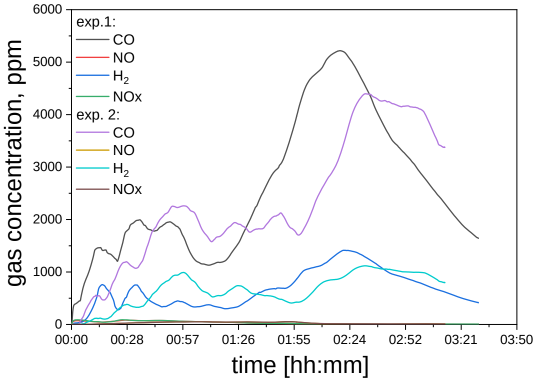

2(a): TOS in stove 1 (CO, NO, H_2, NOx)

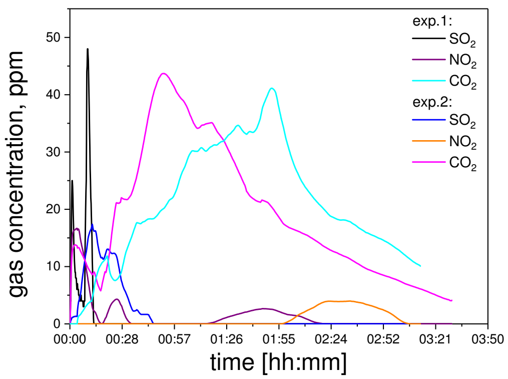

2(b): TOS in stove 1 (SO_2, NO_2, CO_2)

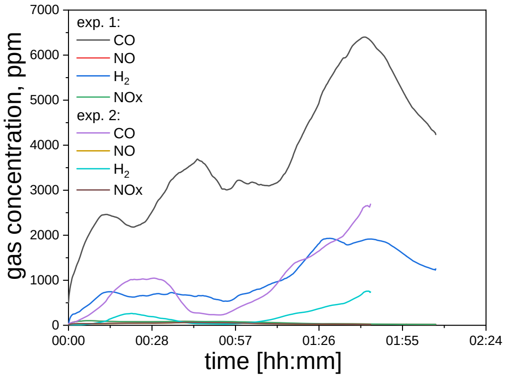

2(c): TOS in stove 2 (CO, NO, H_2, NOx)

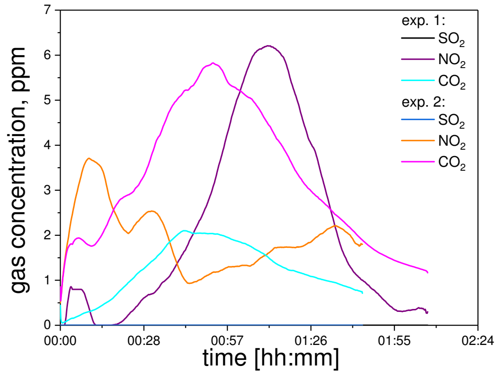

2(d): TOS in stove 2 (SO_2, NO_2, CO_2)

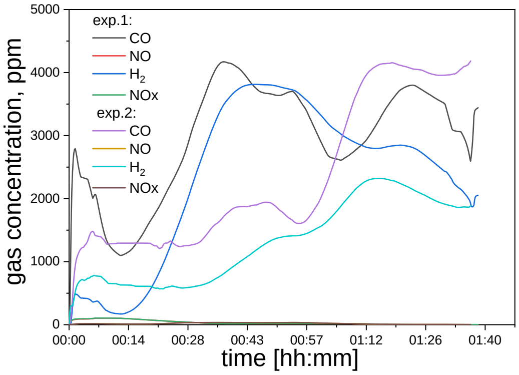

2(e): Peat (CO, NO, H_2, NOx)

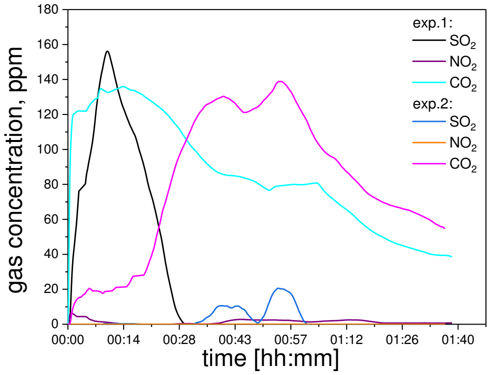

2(f): Peat (SO_2, NO_2, CO_2)

Figure S-2: Gas composition (CO, NO, H_2, NOx, SO_2, NO_2, CO_2) during combustion of torrefied olive stones briquettes in conventional or Ecodesign stove and peat sod in the conventional stove.

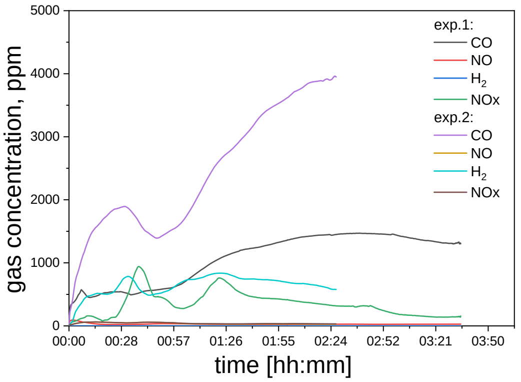

3(a): Ecobrite in conventional (CO, NO, H_2, NOx)

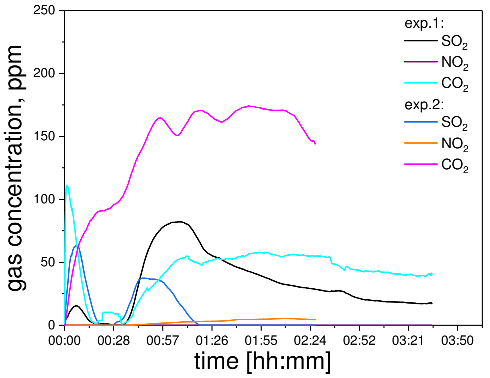

3(b): Ecobrite in conventional (SO_2, NO_2, CO_2)

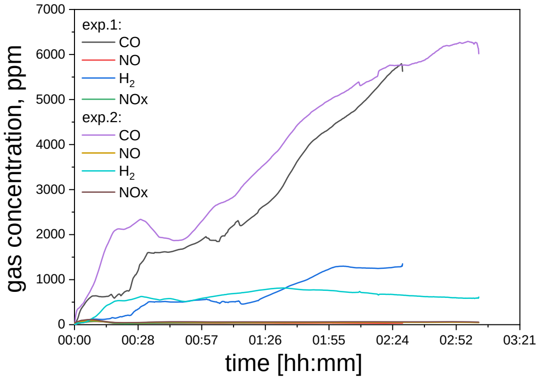

3(c): Ecobrite in Ecodesign (CO, NO, H_2, NOx)

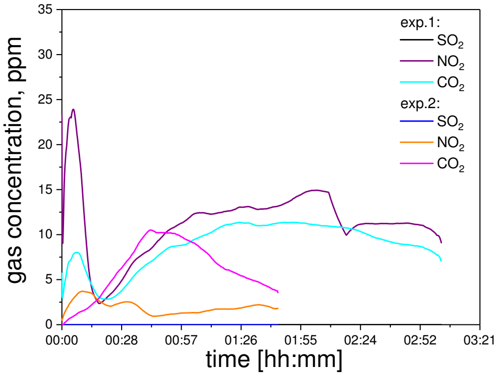

3(d): Ecobrite in Ecodesign (SO_2, NO_2, CO_2)

Figure S-3: Gas composition (CO, NO, H_2, NOx, SO_2, NO_2, CO_2) during combustion of Ecobrite briquettes using only primary air supply in conventional and Ecodesign stoves.

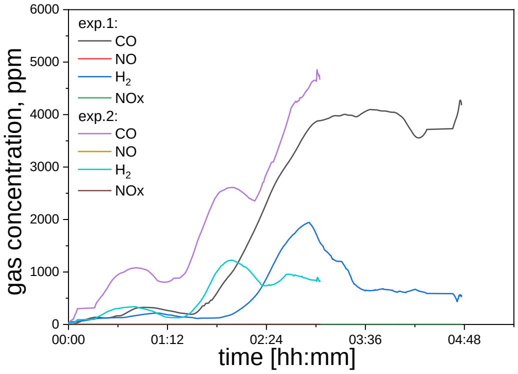

4(a): Smoky coal in conventional (CO, NO, H_2, NOx)

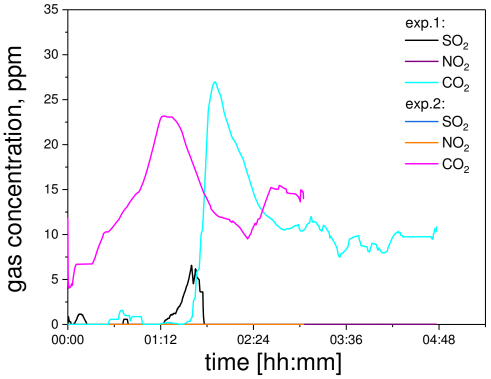

4(b): Smoky coal in conventional (SO_2, NO_2, CO_2)

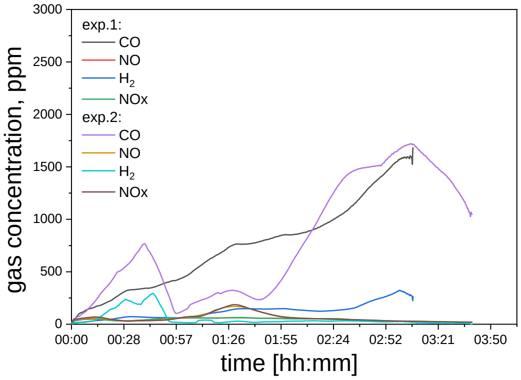

4(c): Smoky coal in Ecodesign (CO, NO, H_2, NOx)

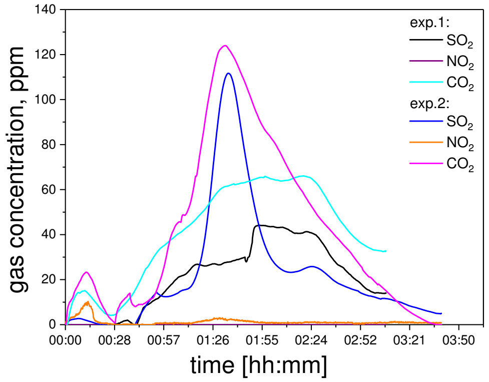

4(d): Smoky coal in Ecodesign (SO_2, NO_2, CO_2)

Figure S-4: Gas composition (CO, NO, H_2, NOx, SO_2, NO_2, CO_2) during combustion of smoky coal using only primary air supply in conventional and Ecodesign stoves.

S-3 CO and CO_2 emission factors

The CO and CO_2 emission factors were calculated for wood logs and torrefied olive stone briquettes using the gas concentrations from TESTO instrument recordings. This study includes the measurements of CO and CO_2 concentrations from burning of wood logs and TOS briquettes because the measurement uncertainty for other fuels was significantly greater than any permitted limits in the literature.

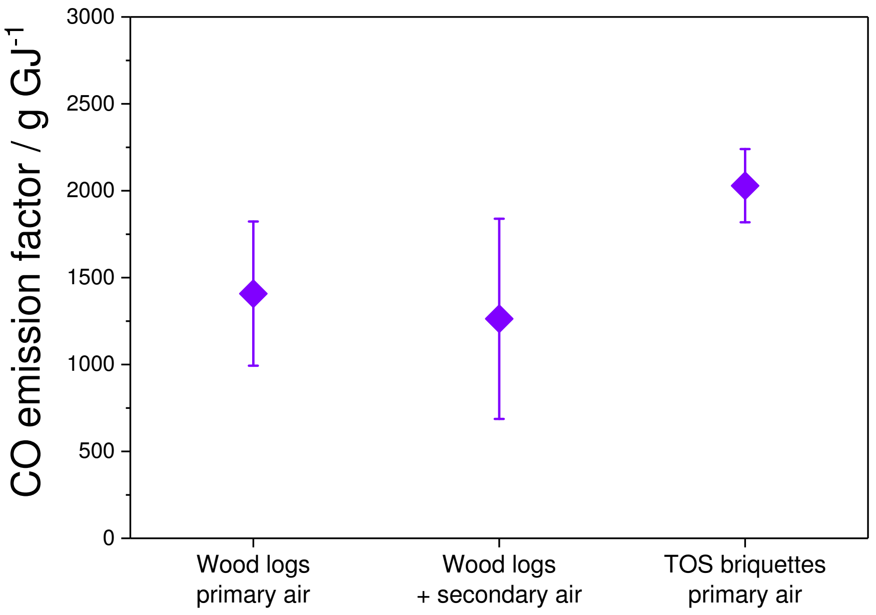

5(a): CO emission factors

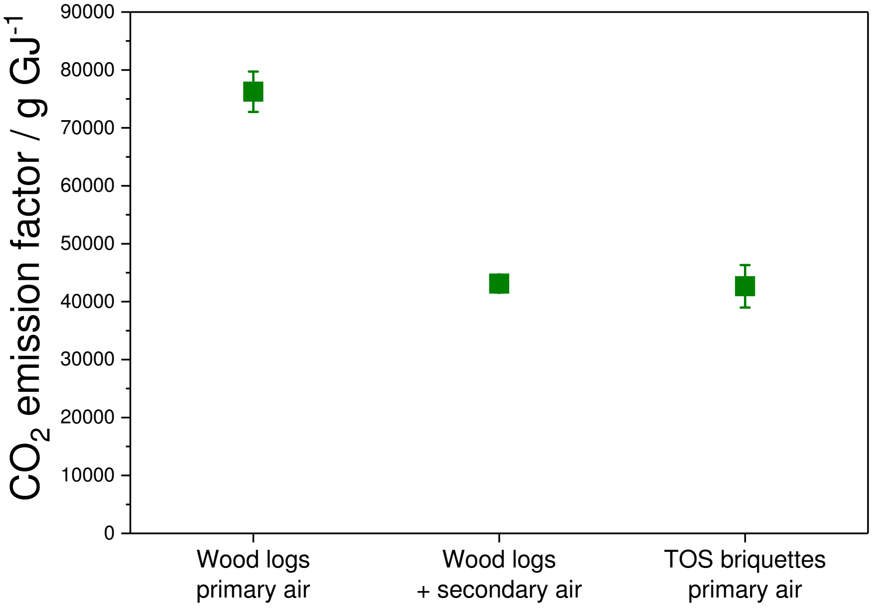

5(b): CO_2 emission factors

Figure S-5: CO and CO_2 emission factors calculated using TESTO analysis data from burning of wood logs using only primary air supply or with the addition of secondary air and torrefied olive stone (TOS) briquettes using primary air only and shown in g GJ^{-1}.

Figure 5(b) showed the CO_2 EFs ($\approx$ 78000 g GJ^{-1}) for the wood stove burning were less than the CO_2 EFs in the literature (107000 g GJ^{-1}) [1]. However, the CO_2 EF for TOS

briquettes (≈ 42600 g GJ^{-1}) were found in this study to be substantially less than CO_2 EFs from wood logs burning.

Moreover, the secondary air supply led to the CO_2 EFs (≈ 43 g MG^{-1}) decrease during wood logs burning compared to that using only primary air, as previously reported by Coffey et al. [2]. Secondary air addition to the wood logs burning is a way to both cool the flue gas and increase the volume, thus decreasing CO_2 emission factors. The values of CO emission factors varied from 600 to 2200 g GJ^{-1} for stove burning of all fuels. However, the CO emission factors were in a range as previously reported in the literature [3,4].

S-4 Thermal efficiency

The thermal efficiency and heat output were calculated for two experiments in Tables S-3-S-4. The values correspond to the calculations and results in the main manuscript (Section 3.8).

Table S-3: Thermal efficiencies of solid fuel-stove combination using primary air or with the addition of secondary air with wood logs for the experiment 1 in conventional and Ecodesign stoves. Wood logs (WL), torrefied olive stones briquettes (TOSB), peat, Ecobrite briquettes (EB), smoky coal (SC) and firelighter (FL) were burned in stoves.

Properties	WL	TOSB	Peat	EB	SC	TOSB	EB	WL
	primary air							+ secondary air
	Conventional					Ecodesign		Conventional
Mass of the test fuel, kg	3.6	3.6	3.8	3.5	4.0	3.5	3.5	3.8
Solids passing the grate, kg	0.1	0.18	0.59	1.6	2.6	0.1	1.9	0.01
Undergrate mass, %	1.0	4.1	3.5	4.7	4.9	3.8	4.9	1.0
Carbon content, kg	1.9	2.21	2.1	2.5	3.1	2.2	2.5	2.0
Flue gas temperature, °C	219.0	210.9	193.7	71.0	244.0	202.6	125.6	161.4
Inlet, l s^{-1}	4.1	4.1	4.7	4.2	3.7	3.6	3.6	3.8
Volume, m^3	39.8	46.1	36.3	160.7	21.1	27.0	31.5	66.1
Time, s	9700	11230	7690	38270	5730	7500	8760	12170
Mass, kg	47.7	55.3	43.3	192.9	25.3	32.4	37.8	79.3
Mass offgas, kg	51.2	58.7	46.5	194.8	26.7	35.8	39.4	83.1
Energy input, kJ	51772	72961	48590	100261	113684	70935	100261	54648
Thermal efficiency, %	72	73	69	81	87	82	89	69
Flue losses, %	26	20	22	13	7	12	6	29
Unburned losses, %	2	7	9	5	6	6	6	2
P, kW	5.2	6.2	5.3	1.4	6.9	9.2	5.2	4.5

Table S-4: Thermal efficiencies of solid fuel-stove combination using primary air or with the addition of secondary air with wood logs for the experiment 2 in conventional and Ecodesign stoves. Wood logs (WL), torrefied olive stones briquettes (TOSB), peat, Ecobrite briquettes (EB), smoky coal (SC) and firelighter (FL) were burned in stoves.

Properties	**WL**	**TOSB**	**Peat**	**EB**	**SC**	**TOSB**	**EB**	**WL**
	**primary air**							**+ secondary air**
	**Conventional**					**Ecodesign**		**Conventional**
Mass of the test fuel, kg	3.2	4.0	4.0	3.6	4.4	3.5	3.6	3.9
Solids passing the grate, kg	0.2	0.2	0.6	2.2	1.6	0.3	1.4	0.01
Undergrate mass, %	1.5	3.6	3.5	4.7	4.3	3.8	4.9	1.0
Carbon content, kg	1.7	2.5	2.2	2.6	3.4	2.2	2.6	2.0
Flue gas temperature, °C	213.2	210.9	244.7	141.0	148.7	198.5	119.5	152.9
Inlet, l s^{-1}	4.7	4.1	4.7	4.2	3.7	3.6	3.6	3.8
Volume, m^3	40.7	49.4	24.1	95.8	36.0	25.9	41.4	73.0
Time, s	8620	12040	9790	22820	9790	7200	11500	13450
Mass, kg	48.8	59.3	43.3	123.7	43.2	31.1	49.7	87.6
Mass offgas, kg	51.8	63.0	46.8	125.1	46.0	34.4	51.8	91.5
Energy input, kJ	46019	81068	51692	103126	125052	70935	101693	56086
Thermal efficiency, %	68	74	64	75	89	82	88	69
Flue losses, %	29	20	27	19	6	11	7	29
Unburned losses, %	3	6	9	5	5	6	6	2
P, kW	5.0	6.4	4.5	1.8	8.1	9.2	5.4	4.2

References

(1) Zijlema PJ, The Netherlands: list of fuels and standard CO_2 emission factors version of April 2015, Report 105822/BL2015. *Netherlands Enterprise Agency* **2015**, 1–6.

(2) Coffey ER,; Muvandimwe D,; Hagar Y,; Wiedinmyer C,; Kanyomse E,; Hannigan MP and etc., New Emission Factors and Efficiencies From in-Field Measurements of Traditional and Improved Cookstoves and Their Potential Implications. *Environ Sci Technol* **2017**, *51*, 12508–17.

(3) Fleming LT,; Weltman R,; Yadav A,; Edwards RD,; Arora NK,; Smith KR and etc., Emissions from village cookstoves in Haryana, India, and their potential impacts on air quality. *Atmos Chem Phys* **2018**, *18*, 15169–82.

(4) Mitchell EJS,; Cottom J,; Phillips D,; Dooley B, A review of the impact of domestic combustion on UK air quality. *Independent report commissioned for HETAS* **2019**, 1–108.
